# Discharging a Li-S battery with ultra-high sulphur content cathode using a redox mediator

**DOI:** 10.1038/srep32433

**Published:** 2016-08-30

**Authors:** Kwi Ryong Kim, Kug-Seung Lee, Chi-Yeong Ahn, Seung-Ho Yu, Yung-Eun Sung

**Affiliations:** 1 Center for Nanoparticle Research, Institute for Basic Science (IBS), Seoul 151-742, South Korea; 2 School of Chemical and Biological Engineering, Seoul National University, Seoul 151-742, South Korea; 3 Beamline Department, Pohang Accelerator Laboratory (PAL), Pohang 790-784, South Korea

## Abstract

Lithium-sulphur batteries are under intense research due to the high specific capacity and low cost. However, several problems limit their commercialization. One of them is the insulating nature of sulphur, which necessitates a large amount of conductive agent and binder in the cathode, reducing the effective sulphur load as well as the energy density. Here we introduce a redox mediator, cobaltocene, which acts as an electron transfer agent between the conductive surface and the polysulphides in the electrolyte. We confirmed that cobaltocene could effectively convert polysulphides to Li_2_S using scanning electron microscope, X-ray absorption near-edge structure and *in-situ* X-ray diffraction studies. This redox mediator enabled excellent electrochemical performance in a cathode with ultra-high sulphur content (80 wt%). It delivered 400 mAh g^−1^_cathode_ capacity after 50 cycles, which is equivalent to 800 mAh g^−1^_S_ in a typical cathode with 50 wt% sulphur. Furthermore, the volumetric capacity was also dramatically improved.

Lithium-sulphur (Li-S) batteries have attracted much recent attention, due to the low cost and high theoretical specific capacity (1672 mAh g^−1^) of sulphur^1^,^2^. They have great potential as energy storage devices for electric vehicles (EVs) and energy storage systems (ESSs)^3^. However, there are several obstacles for their commercialization. One is the presence of polysulphides, which are intermediate products formed during charge/discharge, and their dissolution into the electrolyte leads to continuous loss of the active material and self-discharge^4^,^5^. Past researches have focused on constraining the movement of polysulphides by infiltrating sulphur into meso and/or micro porous carbon materials, physically restricting the dissolution of polysulphides^6^,^7^,^8^,^9^, or designing a surface (e.g., functionalized carbon, metal oxide or metal carbide) that effectively adsorbs polysulphides^10^,^11^,^12^,^13^,^14^. Another major obstacle is the insulating nature of sulphur. In order to compensate for the low electrical conductivity of sulphur (5 × 10^−30 ^S cm^−1^ at room temperature), 30–40 wt% conductive agent is typically needed. This also necessitates the use of a binder (10–20 wt%) to attach the conductive agent and active material onto the current collector. The conductive agent and binder limited the amount of sulphur in the cathode to 50–60 wt%^6^,^7^,^11^,^12^, leading to significantly lowered energy density and specific capacity per cathode mass, as well as increased manufacturing cost. The solution is increasing the effective conductivity of the cathode at high sulphur loading. To the best of our knowledge, all studies reporting high sulphur mass loading in Li-S batteries have achieved it by using thick cathodes^15^,^16^,^17^,^18^. This increases the areal capacity but not the volumetric capacity, even though the latter is more meaningful criterion^19^. Improving the volumetric capacity requires a higher sulphur ratio in the cathode material. Infiltrating sulphur into porous carbon only provides a limited solution, since the increased sulphur ratio is achieved at the cost of reduced conductivity from carbon. Additional conductive agents are needed in these composites, therefore sulphur loading in the cathodes remains low^20^.

In this paper, the effective cathode conductivity is increased by introducing a redox mediator cobaltocene (Co(η^5^C_5_H_5_)_2_) into the electrolyte. It has a redox potential within the region of polysulphide reduction, and therefore can act as an electron transfer agent between the conductive surface and polysulphides. We will show that cobaltocene enabled Li_2_S nucleation and growth not only on the conductive carbon surface but also in the electrolyte. This redox mediator allows us to achieve significant improvement in the electrochemical performance of the cathode with ultra-high sulphur content (80 wt%).

## Results

### Cobaltocene acts as a redox mediator

**Figure 1:**
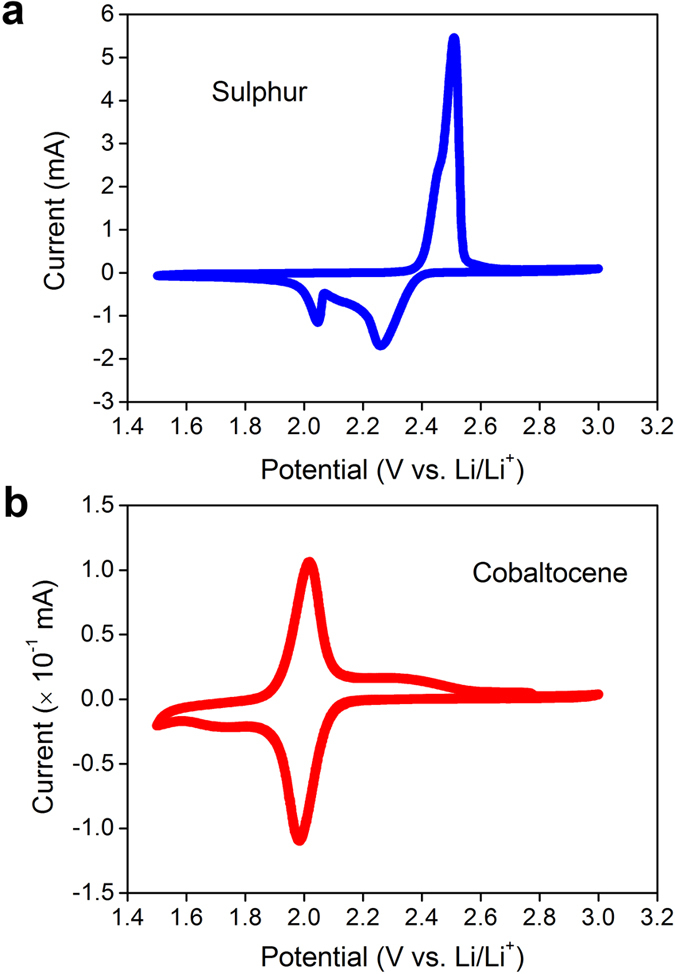
Cyclic voltammograms. (**a**) Sulphur and (**b**) cobaltocene. The sweep rate was 0.2 mV s^−1^.

To measure the redox potentials of sulphur, cyclic voltammogram (CV) test was performed using a sulphur loaded commercial carbon non-woven layer (gas diffusion layer, GDL) as the working electrode, and Li metal as the counter electrode. In Fig. 1a, two pairs of electrochemical redox peaks appear. The high potential reduction peak (2.30 V vs. Li/Li^+^) involves the reduction of sulphur into long-chain polysulphides (Li_2_S_X_, 4 ≤ X ≤ 8). The low potential reduction peak (2.05 V) indicates the reduction of polysulphides to Li_2_S. Note that the area under the 2.05 V reduction peak is smaller than that of the 2.30 V peak, it is opposite from typical behaviour in Li-S batteries^21^,^22^,^23^. It is due to the limited surface area of GDL. A separate CV test was performed in a coin cell with 50 mM cobaltocene dissolved electrolyte, using a bare GDL as working electrode. From Fig. 1b, cobaltocene has a sharp redox potential at 2.00 V, which is lower than the second reduction peak of sulphur (2.05 V). Therefore, in principle, cobaltocene has the potential capability for reducing polysulphides to Li_2_S.

**Figure 2:**
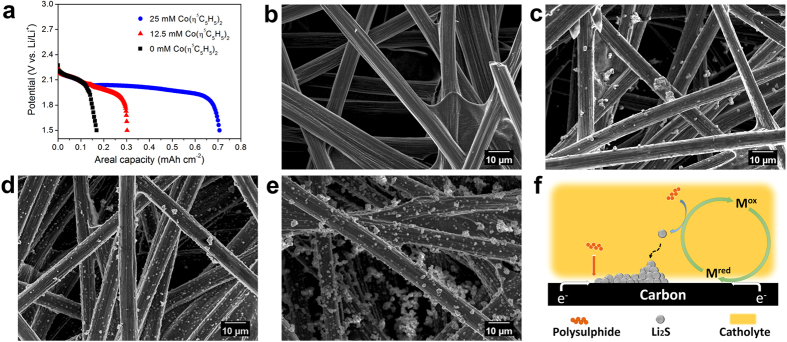
Cobaltocene acts as a redox mediator. (**a**) Galvanostatic discharge curves of cells with different concentrations (0, 12.5, 25 mM) of cobaltocene in 1 M [S] catholyte. SEM images of (**b**) bare GDL and after discharge with (**c**) 0 mM, (**d**) 12.5 mM, and (**e**) 25 mM cobaltocene. (**f**) Schematic illustration of unified mechanism. Li_2_S nucleation and growth through conductive surface pathway and solution pathway with cobaltocene.

In order to test this possibility, we prepared three coin cells with different concentration of cobaltocene (0 mM, 12.5 mM and 25 mM) in 1 M [S] catholyte in Li_2_S_6_ form. The working and counter electrodes were GDL and Li metal, respectively. All tests were performed under galvanostatic discharge conditions with a current density of 500 μA cm^−2^. Since the active material was a polysulphide, there is no high discharge plateau (at about 2.30 V) related to the reduction of sulphur to polysulphides^24^, as shown in Fig. 2a. The discharge capacity increases with cobaltocene concentration. According to the CV results, 50 mM cobaltocene only contributes 30 μAh cm^−2^ to the discharge capacity. Therefore, the increased capacity here is not from cobaltocene itself, but from the low discharge plateau where the polysulphides are reduced to Li_2_S.

### Li_2_S formation

Scanning electron microscope (SEM) images of bare GDL and after the first discharge (Fig. 2b–e) show a large number of highly agglomerated Li_2_S particles when the concentration of cobaltocene is increased. It is a clear evidence that cobaltocene effectively reduces the polysulphides to solid Li_2_S. Typical Li-S batteries have a potential plateau at 2.1–2.0 V after a gently sloping region between 2.3–2.1 V. However, this plateau almost disappeared in the absence of cobaltocene. This can be understood from the magnified SEM images. The surface of bare carbon fiber was clear and smooth (Supplementary Fig. 1a), but completely covered with Li_2_S particles after discharge (Supplementary Fig. 1b). Since Li_2_S is insoluble in the electrolyte and has high electrical resistivity, electron transfer from conductive carbon surface to the Li_2_S/electrolyte interface incurs high polarization^25^. More specifically, the limited conductive surface area cannot support enough sites to accommodate Li_2_S, leading to the characteristic potential drop and limited discharge capacity. The redox mediator acts as an electron transfer agent between conductive surface and polysulphides: it is reduced directly at the carbon fiber surface to M^red^, which diffuses into the electrolyte, then reduce the polysulphides there while itself is oxidized back to M^ox^. Hence the redox mediator allows Li_2_S nucleation and growth on the carbon surface and in the electrolyte simultaneously. Afterwards, Li_2_S nanoparticles in the electrolyte will attach to and agglomerate with those already on the carbon surface to reduce the surface energy (Fig. 2f). As a result, cobaltocene in the electrolyte allows not only more Li_2_S formation but also a thicker Li_2_S layer (Supplementary Fig. 1c). We also quantitatively demonstrated it using energy-dispersive X-ray spectroscopy (EDS) analysis (Supplementary Fig. 2).

**Figure 3:**
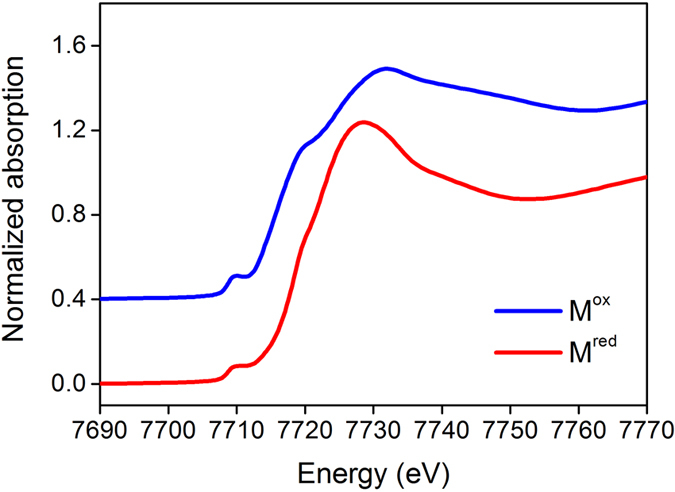
Chemical states of redox mediators. Normalized Co K-edge XANES spectra for M^red^ and M^ox^.

In order to confirm that the cobaltocene reduces polysulphides by being oxidized into cobaltocenium, we conduct X-ray absorption near-edge structure (XANES) analysis. Figure 3 shows the normalized Co K-edge XANES spectra for M^red^ (0.5 M cobaltocene in electrolyte) and M^ox^ (obtained by adding polysulphides into M^red^ and stirring for 6 h) at energies corresponding to the electronic transition from Co 1s to 4p. There are three peaks in the rising edge region. The pre-edge peak at 7710 eV corresponds to electric dipole-forbidden (1s → 3d) transition, which is enabled by 4p–3d hybridization due to the distortion of local structure. The absorption peak at 7720 eV is assigned to 1s → Cp (π*) transition (Cp is cyclopentadienyl). The strong peak at the top of the edge is 1s → 4p transition (7730 eV)^26^,^27^,^28^. As the reaction proceeds, the 1s → 4p transition energy (above 7720 eV) increases and the peak shifts from 7728 to 7732 eV, presenting clear evidence of the oxidation of Co^2+^ (cobaltocene) into Co^3+^ state (cobaltocenium)^29^. In addition, the intensities of pre-edge peak and the shoulder on the rising edge are increased. Similar results have been reported for other organometallic compounds (e.g., ferrocenium, the oxidized state of ferrocene)^30^.

**Figure 4:**
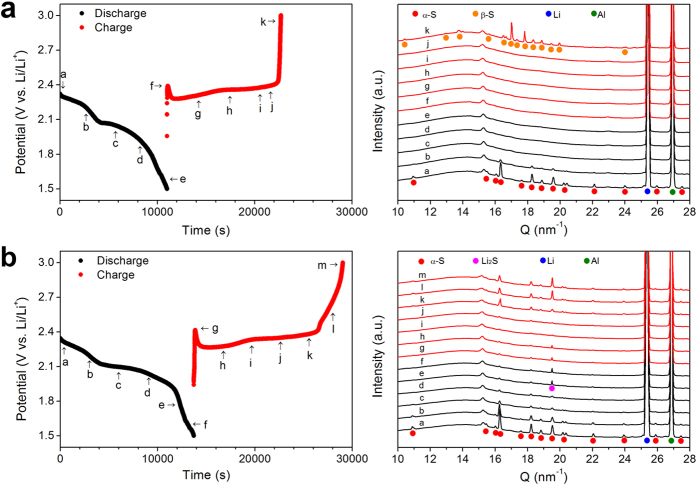
Galvanostatic discharge-charge curves of the first cycle and *in-situ* XRD patterns for points labeled on the curves. (**a**) Without cobaltocene, and (**b**) with 50 mM cobaltocene. Only the XRD patterns with cobaltocene show the appearance of Li_2_S peaks at the end of discharge.

To further verify that the increased capacity is from Li_2_S formation, we conducted *in-situ* X-ray diffraction (XRD) analysis during the first cycle with and without 50 mM cobaltocene. A cathode with 70 wt% S, 15 wt% carbon and 15 wt% binder was used as the working electrode. The cells were galvanostatic charged/discharged at 200 μA cm^−2^. Without cobaltocene, the intensities of orthorhombic α-sulphur (PDF no. 00-008-0247) peaks gradually decreased during the initial discharge and completely disappeared after the high potential region (Fig. 4a). No additional identical peak was found until the end of the discharge. It suggests that there is no detectable crystalline Li_2_S. Nevertheless, the gradually sloping (as opposed to a plateau) discharge curve at the low potential region is due to a thin insulating Li_2_S layer covering the conductive carbon surface and hindering charge transfer. As explained earlier, this layer leads to limited discharge capacity from the low potential plateau. With cobaltocene, in contrast, there is a clear discharge potential plateau for Li_2_S formation. XRD patterns also display Li_2_S (111) Bragg peak at 19.5 nm^−1^ (PDF no. 00-023-0369) towards the end of discharge (Fig. 4b). It could be interpreted that more and larger Li_2_S particles were formed in the presence of cobaltocene. The complete disappearance of the Li_2_S peak during the charging process means that the thick Li_2_S layer formed through solution and surface reactions is effectively converted to polysulphides and then to sulphur. The Li_2_S particles in direct contact with the conductive surface can be easily charged. However, their insulating nature inhibits the charging of Li_2_S particles away from the conductive surface. Cui’s group reported polysulphides could act as a redox mediator during the charging process^31^. Our group also reported similar result recently^32^. A small amount of polysulphides in electrolyte could effectively reduce the charging overpotential. This is also the case here. The long chain polysulphides migrate to Li_2_S and produce short chain polysulphides through disproportionation reaction. The short chain polysulphides could dissolve into the electrolyte and participate in the charging process.

### Battery cycling for ultra-high sulphur content cathode

**Figure 5:**
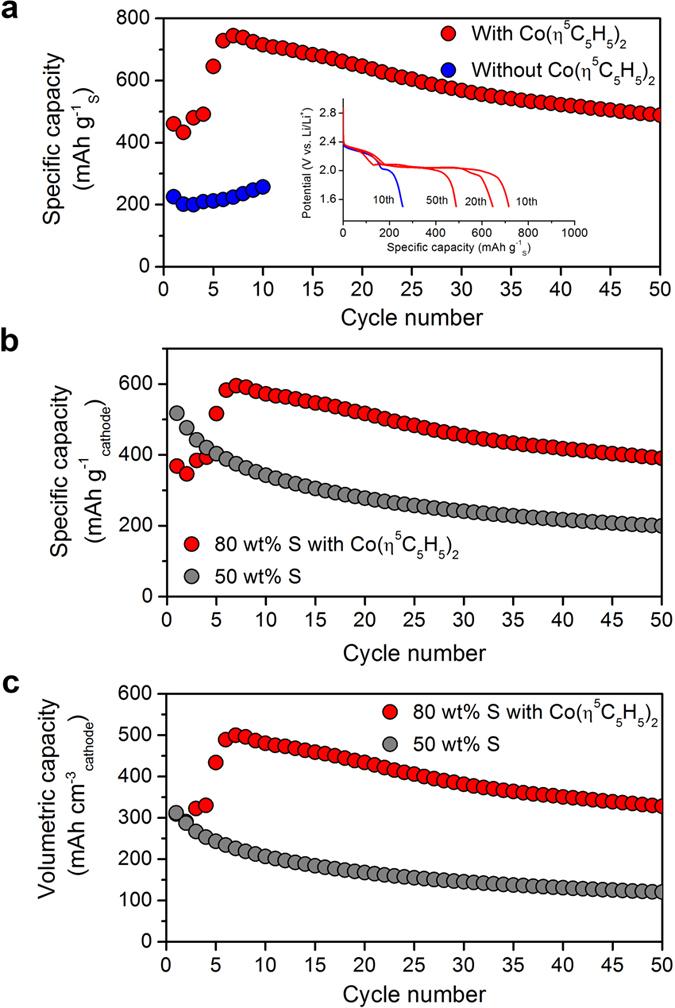
Electrochemical performance. (**a**) Electrochemical characterization of ultra-high sulphur content (80 wt%) cathode with and without cobaltocene. The cycle performance shows dramatically improved discharge capacity with cobaltocene. The inset shows glavanostatic discharge curves of various cycles. (**b**) Specific capacities based on cathode and (**c**) volumetric capacities versus cycle number of 80 wt% S cathode with cobaltocene and 50 wt% S cathode. All tests were performed at 0.1 C.

The results discussed so far show that cobaltocene can transfer electrons from electrode to the polysulfieds. This could compensate for the limited conductivity of the cathode with extremely low conductive agent ratio. A cathode with 80 wt% sulphur, 10 wt% carbon and 10 wt% binder was prepared by simple mortar mixing method. When cells using this cathode were cycled at 0.1 C (167.2 mA g^−1^_S_) with and without 50 mM cobaltocene, the initial discharge capacities were dramatically different, as seen in Fig. 5a. After several cycles, the cell with cobaltocene displayed a capacity of about 750 mAh g^−1^_S_, while the reference cell delivered only 250 mAh g^−1^_S_, which was mainly from the high potential plateau region (inset of Fig. 5a). Moreover, there was abrupt change in voltage during charging process (see Supplementary Fig. 3). We showed initial several cycle data due to the unstable charging behaviour without cobaltocene. The slightly increased capacity might be due to the wetting process as reported before^33^. Correspondingly, increase of capacity during initial several cycles with cobaltocene was observed, and this was due to the activation process of cobaltocene as redox mediator. We also confirmed the reproducibility of these electrochemical properties with longer cycle number as showed in Supplementary Fig. 4. As explained earlier, the low capacity without cobaltocene is due to the limited amount of conductive carbon, which not only transfers electrons but also provides sites for Li_2_S nucleation and growth. The redox mediator cobaltocene acts as a liquid conductive agent. It effectively transfers electrons from the cathode to the polysulphides, and therefore allows Li_2_S nucleation and growth on and off the conductive framework.

Currently, the typical cathode in Li-S batteries contains only 50 wt% sulphur. Therefore, although its specific capacity based on the sulphur mass is high, the value based on total cathode mass is very low. The measured value of 200 mAh g^−1^ per cathode mass after 50 cycles (Fig. 5b) is similar to that of commercial LiCoO_2_ cathode^1^. Our cathode with 80 wt% sulphur using cobaltocene shows 200% increased capacity after 50 cycles. We also shown the discharge capacity based on cathode volume at Fig. 5c. The volumetric capacity is dramatically improved due to the increased sulphur content in cathode.

The rate capability performance with cobaltocene is shown in Supplementary Fig. 3. The thick layer of large Li_2_S particles on the conducive surface after discharge significantly hinders the following charging process. Even though the polysulphides could act as a redox mediator as described earlier, they are far less effective than cobaltocene, especially at high current density. Still, the cell was functional and delivered about 200 mAh g^−1^_S_ capacity at a high current density of 1.2 A g^−1^_S_. When the charging current was fixed to 0.2 A g^−1^_S_ from the 6th cycle on (slow charging), the rate capability improved remarkably. It delivers about 350 mAh g^−1^_S_ capacity at 1.2 A g^−1^_S_ current density. Therefore, we believe that the rate capability could be further improved with another redox mediator that could effectively recharge Li_2_S.

## Conclusion

In summary, we have successfully realized the high performance Li-S battery for ultra-high sulphur content (80 wt%) cathode by using cobaltocene as a redox mediator in the electrolyte. The redox mediator acts as an electron transfer agent: it is reduced at the cathode and then oxidized by the polysulphides remote from the conductive surface to produce Li_2_S. This novel approach can effectively produce Li_2_S both on the conducting surface and in the solution. Taken together, this unified mechanism allows sufficient Li_2_S formation with a very low amount of conductive agent in the cathode, as confirmed by our electrochemical method, SEM, XANES and *in-situ* XRD studies. The results reported here provide a simple and scalable approach to one of the most important challenges in creating ultra-high sulphur content cathodes for Li-S batteries.

## Methods

### Synthesis

The blank electrolyte consists of 1 M lithium bis(trifluoromethanesulfone)imide (LiTFSI) in a mixture of 1,2-dimethoxyethane (DME) and 1,3-dioxolane (DOL) at a 1:1 volume ratio, with 0.1 M LiNO_3_ as an additive. To prepare the polysulphide catholyte, sulphur powder (Alfa Aesar) and lithium sulphide (Alfa Aesar) in fixed ratio were added to the blank electrolyte to achieve 1 M sulphur concentration in the form of Li_2_S_6_. The catholyte was heated at 45 °C for 24 h. A designated amount of cobaltocene was prepared at room temperature after stirring for 12 h. All processes were performed in an Ar-filled glove box.

Sulphur loaded commercial carbon non-woven layer (GDL, Toray) was prepared by dropping sulphur dissolved CS_2_ solution onto GDL electrode.

### Characterizations

X-ray absorption near-edge fine structure (XANES) was measured at 8C nano-probe XAFS beamline (BL8C) of Pohang Light Source (PLS-II) in the 3.0 GeV storage ring with a ring current of 360 mA. The radiation source of BL8C is a tapered in-vacuum-undulator. The X-ray beam was monochromated by a Si(111) double crystal and then it was delivered to a secondary source aperture where the beam size was adjusted to be 0.3 mm (v) × 1 mm (h). A high voltage (3000 V) was applied to ionization chambers which were filled with N2/Ar mixture gases to detect x-ray intensity. XAFS measurement was conducted in a transmission mode. The samples were prepared with solvent wetted glass fiber separators. After wetting the glass fibers were covered with Kapton tape. The obtained spectra were processed using Demeter software. In order to align the spectra Co foil was measured simultaneously with the samples.

The *in-situ* XRD analysis was carried out at 5D beamline of the Pohang Light Source. The *in-situ* cells were assembled with specially prepared 2032 coin cells. The coin cells had a Kapton tape window in the center. The morphology and structure of products were characterized with a field-emission scanning electron microscope (FESEM, ZEISS, MERLIN Compact).

### Electrochemistry

The 80 wt% sulphur cathode slurry was created by mixing sulphur, carbon (Super P and multi-walled carbon nanotube (MWCNT) in 1:1 mass ratio) and polyvinylidene difluoride (PVDF) binder with N-Methyl-2-pyrrolidone (NMP) solvent in weight percentages of 80%, 10% and 10%, respectively. The slurry was pasted onto an Al current collector through the doctor blade method, and dried at 60 °C for 12 h. The coated foil was then roll-pressed and cut into 11 mm-diameter disks with a punching machine. The sulphur loading mass was 1.3–1.5 mg cm^−2^. The same procedure was used to prepare the 70 wt% sulphur cathode, except that slurry was made of 70 wt% sulphur, 15 wt% carbon (Super P and MWCNT in 2:1 mass ratio) and 15 wt% PVDF binder. The 50 wt% sulphur cathode was prepared from a 5:3:2 mixture of S, Super P and PVDF.

The volumetric capacity was calculated based on cathode volume (except Al foil). The thickness of cathode was measured using Micrometer measurement.

The 2032 coin-type half cells were assembled using the sulphur cathode and catholyte from above. The counter and reference electrodes were fabricated from lithium foil in an Ar-filled glove box. All electrochemical measurements were carried out using a WBCS3000 cycler (WonATech, Korea) at room temperature. The cyclic voltammogram (CV) tests were performed at a sweep rate 0.2 mV s^−1^ between 3.0 and 1.5 V. All galvanostatic charge/discharge tests were performed between 3.0 and 1.5 V. The C-rates used in this study were based on the mass and theoretical specific capacity of sulphur (i.e., 1672 mAh g^−1^).

## Additional Information

**How to cite this article**: Kim, K. R. *et al*. Discharging a Li-S battery with ultra-high sulphur content cathode using a redox mediator. *Sci. Rep.* **6**, 32433; doi: 10.1038/srep32433 (2016).

## Supplementary Material

Supplementary Information
