# Supplementary material for: Discharging a Li-S battery with ultra-high sulphur content cathode using a redox mediator
Source: Sci Rep. 2016 Aug 30;6:32433. doi: 10.1038/srep32433 (PMC5004098; doi:10.1038/srep32433)
Supplement: Supplementary Information [file srep32433-s1.doc]

**Supplementary Information for**

Discharging a Li-S battery with ultra-high sulphur content cathode using a redox mediator

**Kwi Ryong Kim^1,2^, Kug-Seung Lee^3^, Chi-Yeong Ahn^1,2^, Seung-Ho Yu^1,2^ & Yung-Eun Sung^1,2^**

^1^ Center for Nanoparticle Research, Institute for Basic Science (IBS), Seoul 151-742, South Korea

^2^ School of Chemical and Biological Engineering, Seoul National University, Seoul 151-742, South Korea

^3^ Beamline Department, Pohang Accelerator Laboratory (PAL), Pohang 790-784, South Korea

**Correspondence and requests for materials should be addressed to Y.-E. S. (e-mail: ysung@snu.ac.kr)**


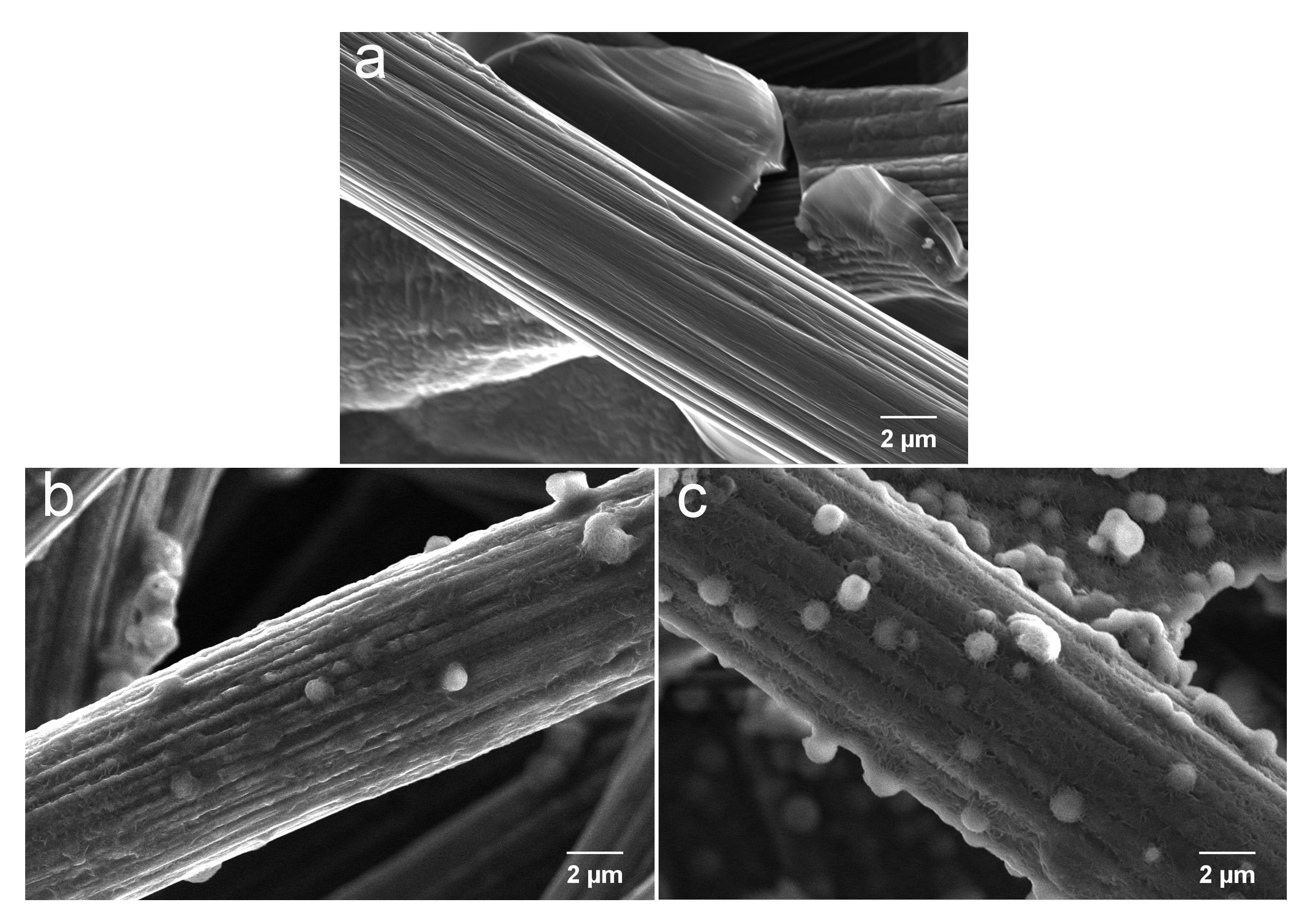


**Supplementary Figure 1.** Magnified SEM images of bare (a) GDL, after discharge with (b) 0 mM, (c) 25 mM cobaltocene in catholyte.


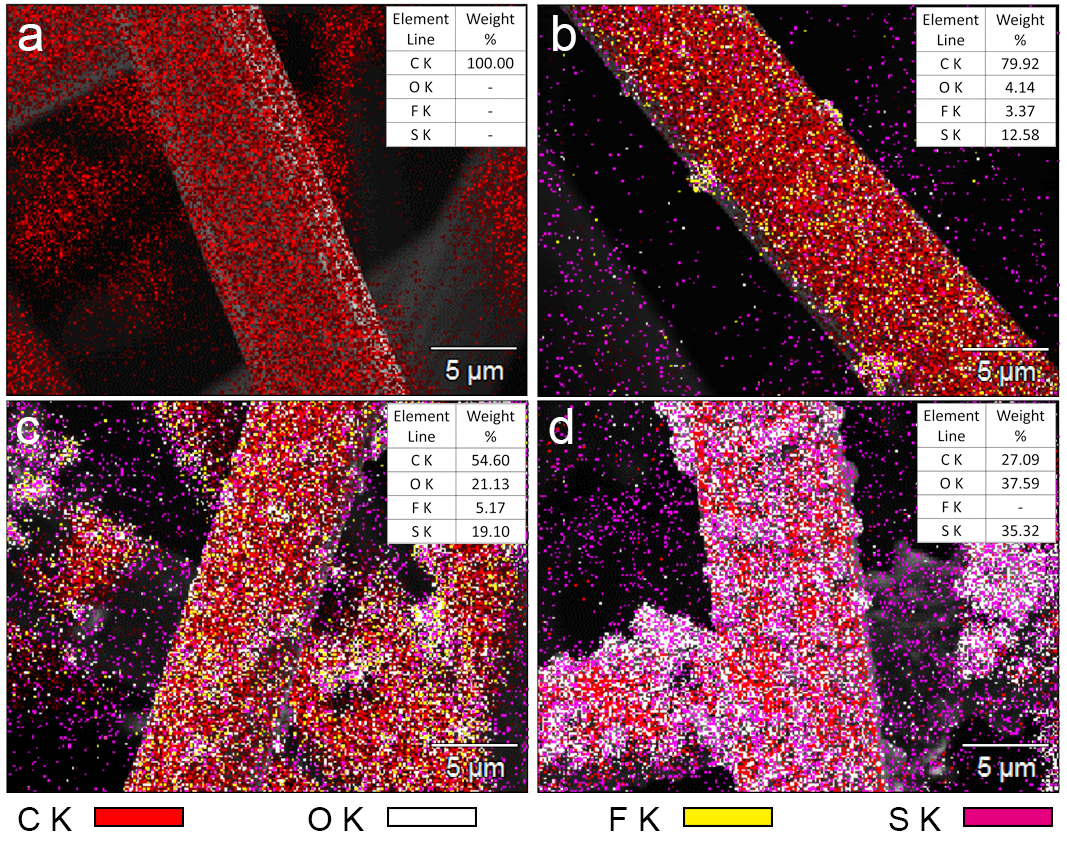


**Supplementary Figure 2.** EDS elemental mapping of (a) bare GDL, after discharge with (b) 0 mM, (c) 12.5 mM, (d) 25 mM cobaltocene. The inset shows the quantitative results.


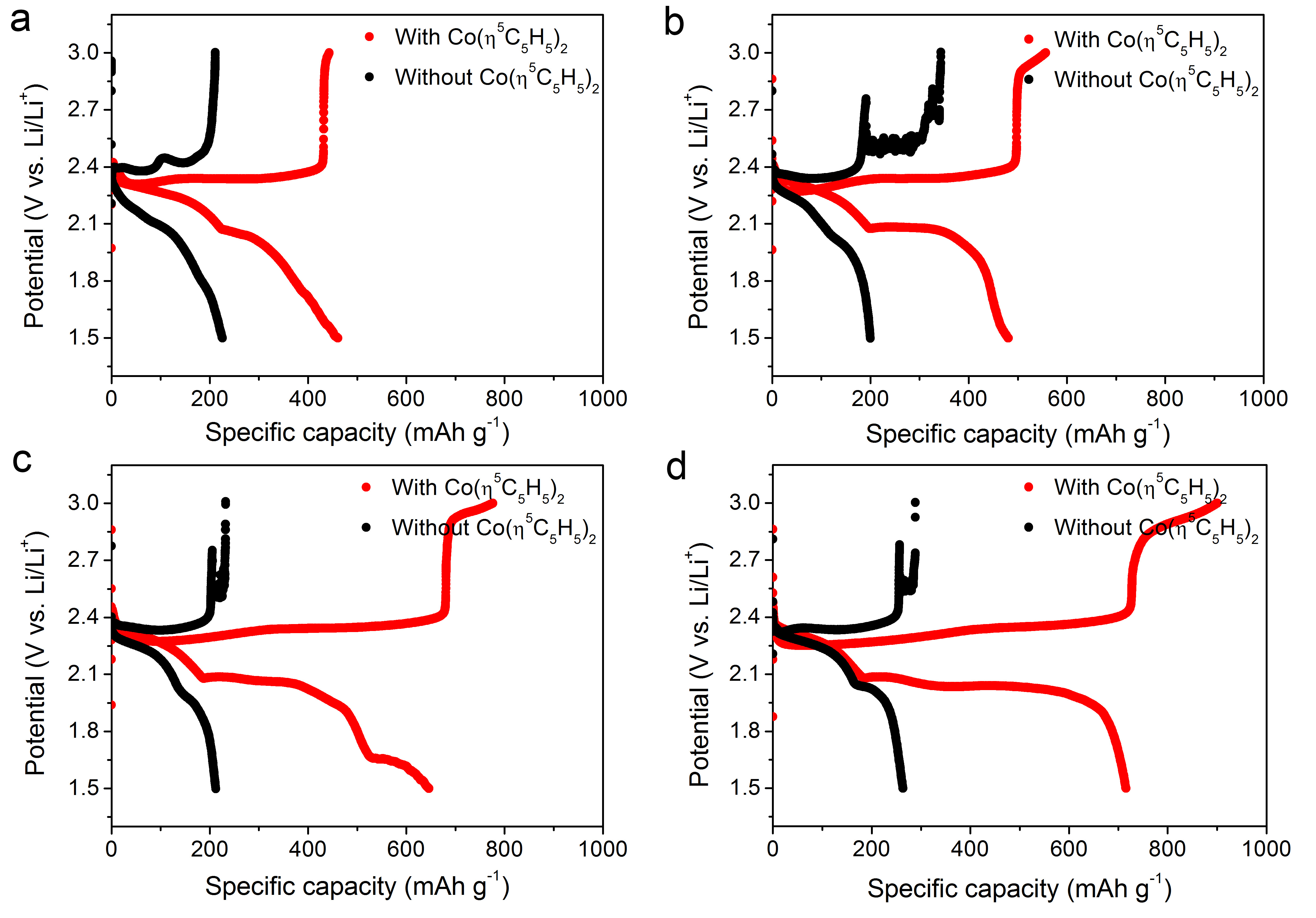


**Supplementary Figure 3.** Galvanostatic charge/discharge curves of (a) 1st, (b) 3rd, (c) 5th, (d) 10th cyc- le with and without cobaltocene.


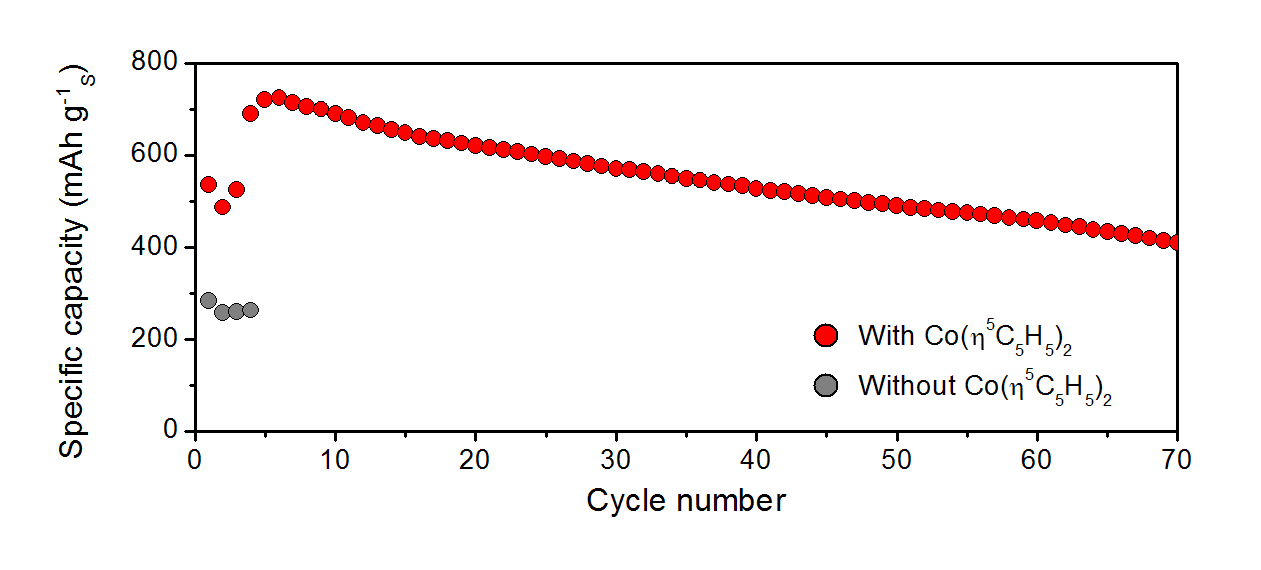


**Supplementary Figure 4.** Cycle performance of ultra-high Sulphur content (80 wt%) cathode with cobaltocene at 0.1 C.


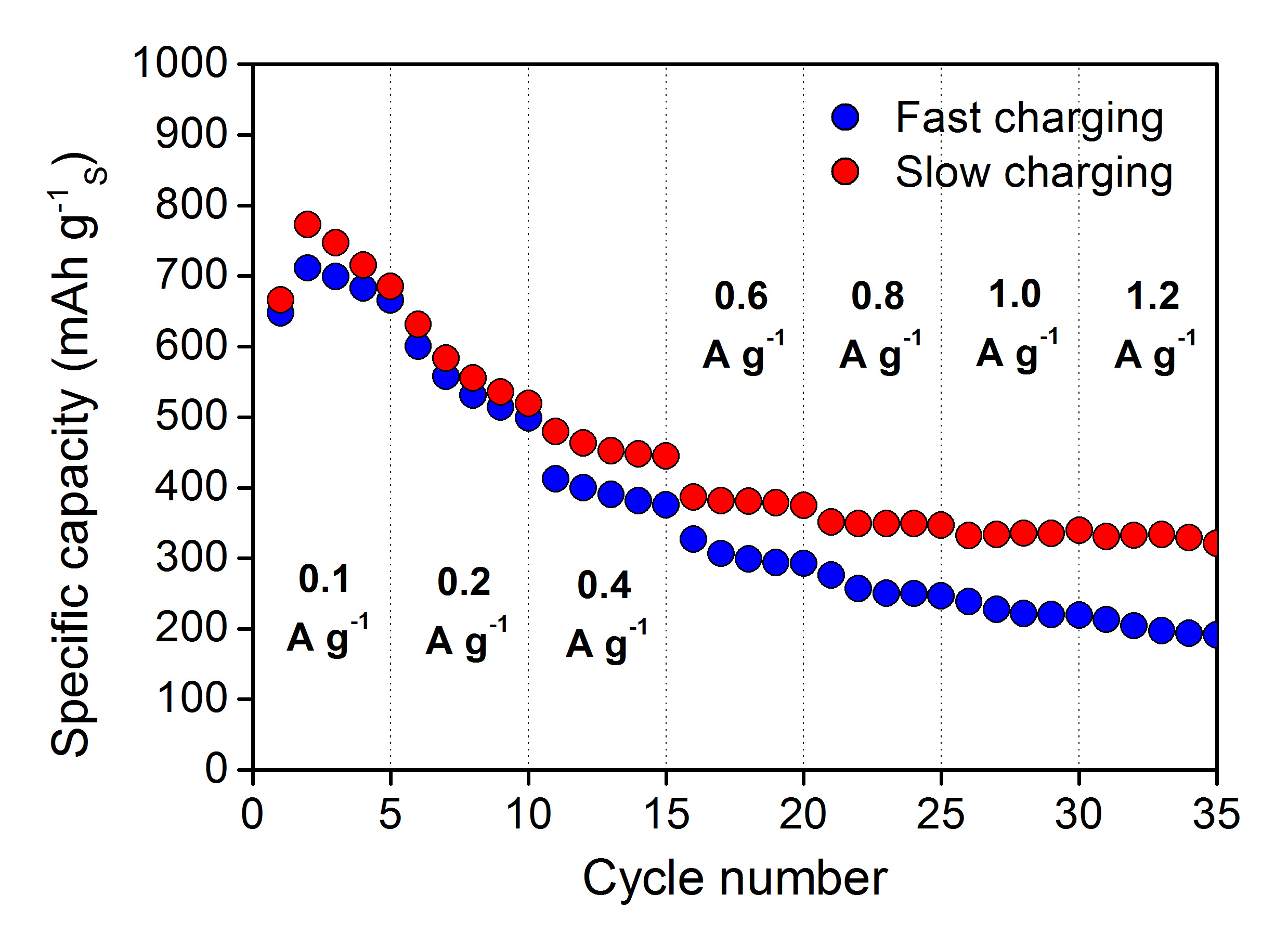


**Supplementary Figure 5.** Rate performances of the cells with different charging current density. The cell is charged/discharged at indicated current density (Fast charging). The other cell is charged at 0.2 A g^-1^ f-rom 6th cycle (Slow charging).
